# Imported and indigenous *Plasmodium Vivax* and *Plasmodium Falciparum* malaria in the Hubei Province of China, 2005–2019

**DOI:** 10.1186/s12936-023-04752-7

**Published:** 2023-11-06

**Authors:** Dongni Wu, Hong Zhu, Lun Wan, Juan Zhang, Wen Lin, Lingcong Sun, Huaxun Zhang, Si Liu, Eimear Cleary, Andrew J. Tatem, Jing Xia, Shengjie Lai

**Affiliations:** 1 Institute of Parasitic Disease Control, Hubei Provincial Center for Disease Control and Prevention, Wuhan, 430079 China; 2 WorldPop, School of Geography and Environmental Science, University of Southampton, Southampton, SO17 1BJ UK

**Keywords:** China, Elimination, Epidemiology, Imported cases, Malaria

## Abstract

**Background:**

The Hubei Province in China reported its last indigenous malaria case in September 2012, but imported malaria cases, particularly those related to *Plasmodium vivax* and *Plasmodium falciparum*, threaten Hubei’s malaria-free status. This study investigated the epidemiological changes in *P. vivax* and *P. falciparum* malaria in this province to provide scientific evidence for preventing malaria resurgence.

**Methods:**

The prevalence, demographic characteristics, seasonal features, and geographical distribution of malaria were assessed using surveillance data and were compared across three stages: control stage (2005–2009) and elimination stages I (2010–2014) and II (2015–2019).

**Results:**

In 2005–2019, 8483 malaria cases were reported, including 5599 indigenous *P. vivax* cases, 275 imported *P. vivax* cases, 866 imported *P. falciparum* cases, and 1743 other cases. Imported *P. falciparum* cases accounted for 0.07% of all cases reported in 2005, but increased to 78.81% in 2019. Most imported *P. vivax* and *P. falciparum* malaria occurred among males, aged 21–60 years, during elimination stages I and II. The number of regions affected by imported *P. falciparum* and *P. vivax* increased markedly in Hubei from the control stage to elimination stage II. Overall, 1125 imported *P. vivax* and *P. falciparum* cases were detected from 47 other nations. Eight imported cases were detected from other provinces in China. From the control stage to elimination stage II, the number of cases of malaria imported from African countries increased, and that of cases imported from Southeast Asian countries decreased.

**Conclusions:**

Although Hubei has achieved malaria elimination, it faces challenges in maintaining this status. Hence, imported malaria surveillance need to be strengthened to reduce the risk of malaria re-introduction.

## Background

Malaria is one of the most serious public health problems worldwide. In 2021, 84 malaria-endemic countries reported 247 million malaria cases and 619,000 malaria-attributed deaths. Most malaria cases (95%) occurred in the African region, followed by the Southeast Asian region (2%) [1]. China has previously experienced severe malaria epidemics [2]. Historically, malaria was endemic in 24 provinces of China, with more than 30 million cases annually reported [3]. Owing to the comprehensive control and prevention strategies implemented in recent decades, the number of reported malaria cases in China has decreased from 61,204 cases in 2006 to 7506 cases in 2010 [4]. The National Malaria Elimination Programme (NMEP) was initiated in 2010, which sought to eliminating indigenous malaria throughout most of the country, with the exception of the Yunnan–Myanmar border areas, by 2015, and to reach a completely malaria-free status across China by 2020 [5]. The “1-3-7” malaria case management model was implemented, i.e., cases reported within 1 day, cases investigated and confirmed within 3 days, and the foci investigation and response within 7 days to interrupt malaria transmission [6]. Following these efforts, malaria cases have decreased markedly in China, and zero indigenous malaria cases have been reported nationwide since 2017 [7].

Hubei Province is situated in central mainland China, it prone to malaria outbreaks in the past, of which *Plasmodium vivax*, *Plasmodium falciparum*, and *Plasmodium malariae* were prevalent, with *P*. *vivax* being the most dominant. After much effort, indigenous *P. falciparum* and *P. malariae* have been eliminated in the early 1960s [8]. No indigenous malaria cases were detected in Hubei province since 2013, which was earlier than countrywide achieved zero indigenous cases (reached since 2017) [7, 9]. In this study, we thus refer to indigenous cases essentially as being related to *P*. *vivax* during 2005–2012 only. Imported malaria cases have increased significantly (9% of all cases in 2010 to 100% of all cases in 2016) [10], particularly among *P. vivax* and *P. falciparum* malaria cases, which threatens Hubei’s malaria elimination status. Similarly, *P. vivax* and *P. falciparum* accounted for the majority of all imported cases in China during 2011–2016 [7, 11]. Imported cases might cause re-establishment of malaria transmission, even in countries or areas that have been malaria-free for many years [12–14]. In addition, *P. falciparum* is the most common species that leads to severe malaria and death [15], therefore, only *P*. *vivax* and *P. falciparum* malaria are main species described in this study.

To address this challenge, an integrated analysis of the epidemiological features of both *P. vivax* and *P. falciparum* malaria has been performed. The evidence of this study could provide a reference for early detection, prevention of malaria re-introduction and for epidemiological surveillance.

## Methods

### Study location

**Fig. 1:**
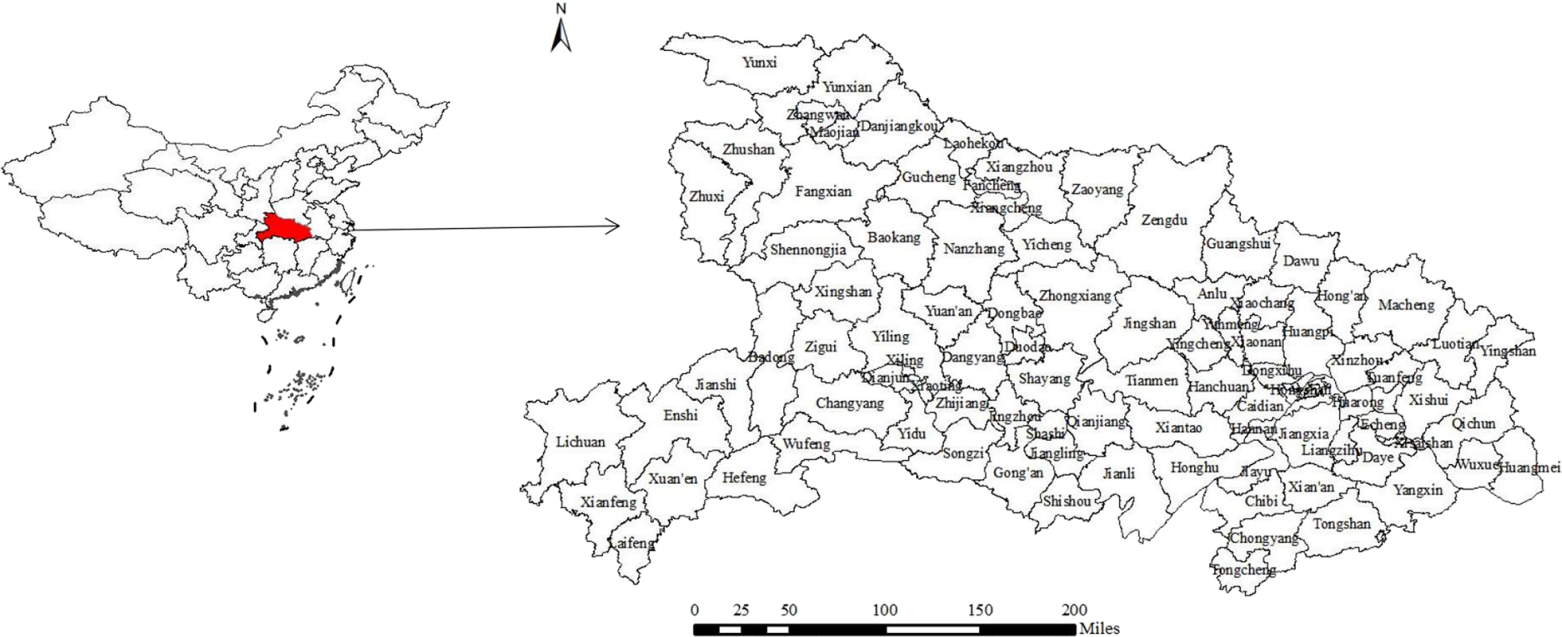
Location of Hubei Province, China

Hubei Province is located in the middle reaches of Yangtze River, and has a subtropical monsoon climate. The environment and climate are conducive to malaria transmission [16]. The area has a population of 57 million and encompasses 185,900 km^2^. Historically, the main malaria vectors in Hubei province was *Anopheles sinensis* and *Anopheles anthropophagus* [17]. No *An. anthropophagus* has been found in Hubei province since 2012 (Fig. 1) [18].

### Data extraction

A retrospective epidemiological study using malaria case data from Hubei Province was conducted for the years 2005–2019. *P. vivax* and *P. falciparum* malaria case data were obtained from the China Information System for Disease Control and Prevention. Data included information on gender, age, address, symptom onset time, reported date, laboratory test result, and travel history of reported cases.

### Case classification

In this study, malaria cases were classified into two categories: clinically diagnosed malaria and laboratory confirmed malaria. A clinically diagnosed case refers to a patient with symptoms of malaria who had a history of travel to known malaria-endemic areas, but without *Plasmodium* detected in the blood. A laboratory confirmed case indicated clinically diagnosed cases with a positive result from any of the following laboratory tests: microscopy, rapid diagnostic tests, or polymerase chain reaction [19]. Based on the Technical Scheme of China Malaria Elimination [20], an indigenous case refers to a malaria infection that occurred via mosquito transmission within the province. An imported case refers to an infection diagnosed in Hubei after being acquired outside of Hubei, including cases from other provinces in China or outside of China. In addition to *Plasmodium falciparum* and *Plasmodium vivax*, the number of other malaria infections, including *Plasmodium ovale*, *Plasmodium malariae*, mixed infections and unclassified cases, was also described.

### Data analysis and visualization

The 15-year study period (2005–2019) was divided into three phases: the control stage (2005–2009) and elimination stages I (2010–2014) and II (2015-–2019). SPSS version 19 (IBM Corp., Armonk, NY, USA) was used to set up a database for this retrospective analysis. ArcGIS version 10.5 (ESRI Inc., Redlands, CA, USA) were used to visualize geographic distribution patterns of *P. vivax* and *P. falciparum* cases in Hubei and the distribution of malaria importation origin by country.

## Results

### Epidemiological profiles of malaria cases

Between 2005 and 2019, 8483 malaria cases were recorded in Hubei, including 5599 indigenous *P. vivax* cases (66.00%), 275 imported *P. vivax* cases (3.24%), 866 imported *P. falciparum* cases (10.21%), and 1743 (20.55%) other cases. The latter included 102 (1.20%) cases of *P. ovale* malaria, 26 (0.31%) cases of *P. malariae* malaria, 3 (0.04%) mixed infection cases, and 1612 (19.00%) unclassified cases (Table 1).

In the control stage (2005–2009), indigenous *P. vivax* cases were found every year. Among all cases, most (5244/6841; 76.66%) were indigenous *P. vivax* malaria. During the elimination stage I (2010–2014), imported *P. vivax* and *P. falciparum* malaria cases presented an increasing trend. In the process of elimination stage II, the majority of imported cases were due to *P. falciparum*. From 2005 to 2013, the number of indigenous *P. vivax* cases dropped markedly from 1171 cases in 2005 to zero in 2013. During the 15-year study period, malaria cases caused by *P. falciparum* increased substantially from 1 (0.07%) in 2005 to 119 in 2019 (78.81%) (Table 1).

**Table 1:** Malaria cases in Hubei Province over 15 years from 2005 to 2019

Stage	Year	*P. vivax*				*P. falciparum*		Others*		Total	
		Indigenous case		Imported case							
Intensified control Stage	2005	1171	(77.14)	4	(0.26)	1	(0.07)	342	(22.53)	1518	(17.89)
	2006	1320	(75.30)	0	(0)	7	(0.40)	426	(24.30)	1753	(20.66)
	2007	1379	(77.95)	36	(2.04)	14	(0.79)	340	(19.22)	1769	(20.85)
	2008	853	(78.33)	5	(0.46)	19	(1.74)	212	(19.47)	1089	(12.84)
	2009	521	(73.17)	13	(1.83)	17	(2.39)	161	(22.61)	712	(8.39)
	Total	5244	(76.66)	58	(0.85)	58	(0.85)	1481	(21.65)	6841	(80.64)
Elimination stage I	2010	266	(62.00)	11	(2.56)	28	(6.53)	124	(28.90)	429	(5.06)
	2011	80	(47.90)	31	(18.56)	55	(32.93)	1	(0.60)	167	(1.97)
	2012	9	(6.82)	44	(33.33)	78	(59.09)	1	(0.76)	132	(1.56)
	2013	0	(0)	32	(24.81)	86	(66.67)	11	(8.53)	129	(1.52)
	2014	0	(0)	21	(15.00)	105	(75.00)	14	(10.00)	140	(1.65)
	Total	355	(35.61)	139	(13.94)	352	(35.31)	151	(15.15)	997	(11.75)
Elimination stage II	2015	0	(0)	9	(7.50)	91	(75.83)	20	(16.67)	120	(1.41)
	2016	0	(0)	21	(13.91)	103	(68.21)	27	(17.88)	151	(1.78)
	2017	0	(0)	16	(16.67)	49	(51.04)	31	(32.29)	96	(1.13)
	2018	0	(0)	17	(13.39)	94	(74.02)	16	(12.60)	127	(1.50)
	2019	0	(0)	15	(9.93)	119	(78.81)	17	(11.26)	151	(1.78)
	Total	0	(0)	78	(12.09)	456	(70.70)	111	(17.21)	645	(7.60)
Total		5599	(66.00)	275	(3.24)	866	(10.21)	1743	(20.55)	8483	(100.00)

*Others include *Plasmodium ovale*, *Plasmodium malariae*, mixed infections, and unclassified cases. The numbers in parentheses in the *Total* column represent the percentage of all cases during 2005–2019, and the numbers in parentheses in the other columns represent the percentage of cases within each year

### Seasonal patterns

**Fig. 2:**
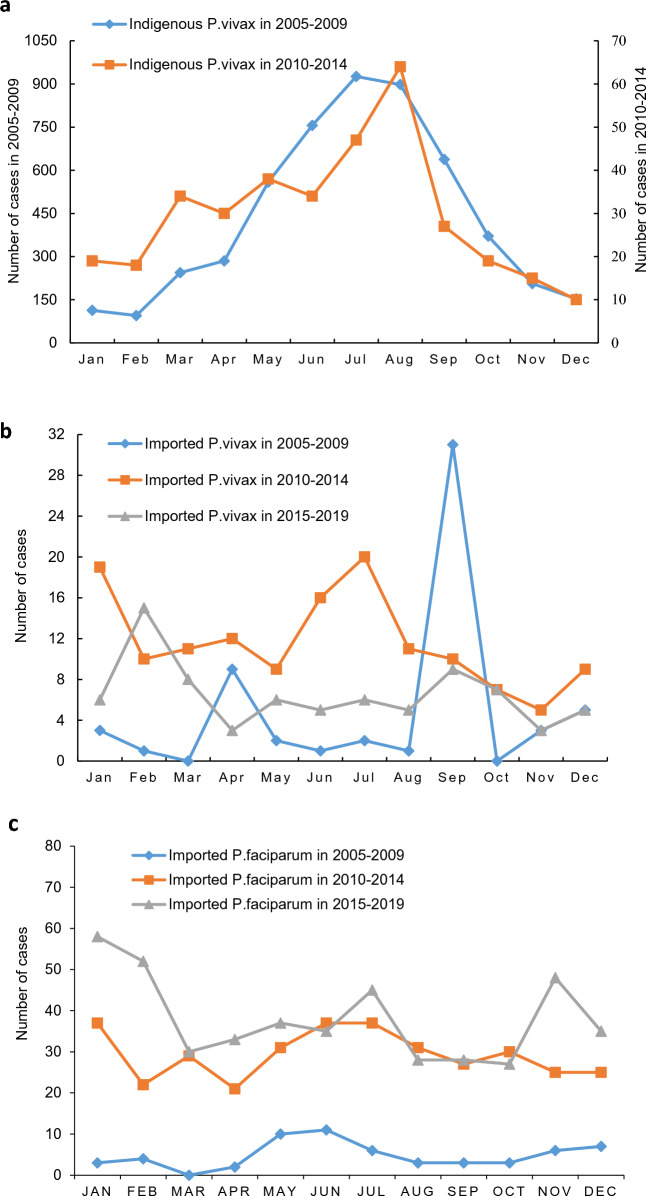
Seasonal patterns of *Plasmodium vivax* and *P. falciparum* malaria cases reported in Hubei province in three phases during 2005–2019. a Indigenous *P. vivax *malaria. b Imported *P. vivax *malaria. c Imported *P. falciparum *malaria

 Among indigenous *P. vivax* cases, seasonal patterns were observed during the control stage (2005–2009) and elimination stage I (2010–2014), where the majority of cases occurred in May to September, with a peak in July and in August, respectively (Fig. 2a). For the three phases of imported *P. vivax* cases, seasonality was thus observed, but the peaks did not occur in the same months. Most cases were reported in April and September during the control stage (2005–2009), with the peak in September. In elimination stage I (2010–2014), two peaks were reached, one in January and one in June–July. In elimination stage II (2015–2019), only one significant peak existed, which occurred in February (Fig. 2b).

Regarding imported *P. falciparum* cases, a similar high peak in June was observed between the control stage and elimination stage I. During elimination stage II (2015–2019), peaks were observed in 3 months, i.e., January, July, and November. Moreover, the highest *P. falciparum* cases in elimination stage I and II both occurred in January (Fig. 2c).

### Demographic characteristics

**Fig. 3:**
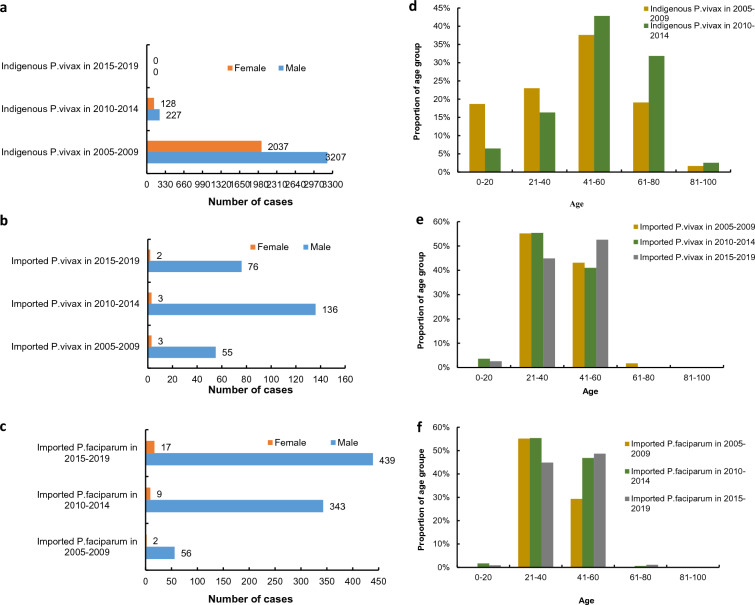
The sex and age distribution of *P. vivax* and *P. falciparum* malaria cases in Hubei province in each stage during a 15-year period.** a** Indigenous *P. vivax *malaria by sex.** b** Imported *P. vivax *malaria by sex.** c** Imported *P. falciparum *malaria by sex.** d** Indigenous *P. vivax *malaria by age group.** e** Imported *P. vivax *malaria by age group.** f** Imported *P. falciparum *malaria by age group

 During the control stage (2005–2009) and elimination stage I (2010–2014), the male-to-female ratios of indigenous *P. vivax* cases were 1.6:1 and 1.8:1, respectively (Fig. 3a). An obvious male predominance was noted among imported *P. vivax* and *P. falciparum* cases. For imported *P. vivax* cases, the male-to-female ratio was 18.3:1 in 2005–2009, 45.3:1 in 2010–2014, and 38:1 in 2015–2019 (Fig. 3b). Regarding imported *P. falciparum* cases, the male-to-female ratios in three stages were 28:1, 38.1:1, and 25.8:1, respectively (Fig. 3c).

As for indigenous *P. vivax*, patient ages ranged from 4 months to 94 years, and 80.35% (4,499/5,599) of the cases occurred in people aged 21–80 years. Moreover, indigenous *P. vivax* cases in elimination stage I was higher than that in the control stage, at the proportion of cases from age 41–80 years. In these three stages, the distribution of age groups of imported *P. vivax* cases was similar to that of imported *P. falciparum* cases. The two age groups with the highest proportions were the 21–40- and 41–60-year age groups, respectively (Fig. 3d, e, f).

### Geographic distribution

**Fig. 4:**
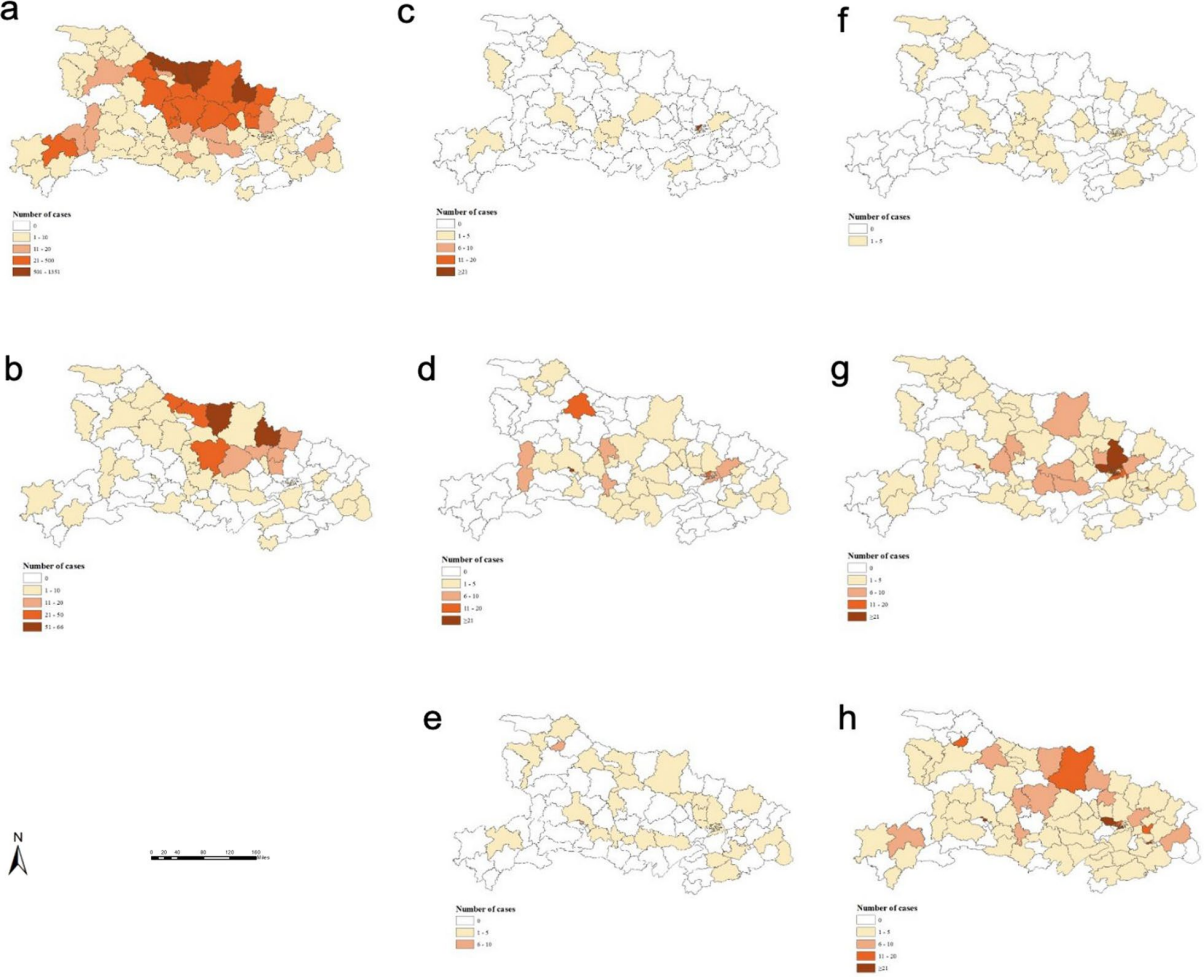
The geographic distribution of *Plasmodium vivax* and *P. falciparum *cases in Hubei province during a 15-year period.** a**,** b** Indigenous *P. vivax* cases in the intensified control stage (2005–2009) and elimination stage I (2010–2014), respectively.** c**,** e** Imported *P. vivax *cases in the intensified control stage (2005–2009), elimination stage I (2010–2014), and elimination stage II (2015–2019), respectively.** f**–**h** Imported* P. falciparum* cases in the intensified control stage (2005–2009), elimination stage I (2010–2014), and elimination stage II (2015–2019), respectively

Indigenous *P. vivax* cases were reported in 82 counties during 2005–2009, and in 42 counties during 2010–2014, respectively. During the control stage (2005–2009) and elimination stage I, 72.35% (4051) of all indigenous malaria cases were reported in Xiangzhou (1386), Zaoyang (1031), Guangshui (816), Laohekou (550), and Zengdu (268) counties. With the rapidly decreasing geographic distribution of indigenous *P. vivax*, none of counties have had an indigenous case of malaria since 2013 (Fig. 4a, b).

The number of counties with imported *P. vivax* cases increased from 14 during 2005–2009 to 42 in 2010–2014, to 36 counties during 2015–2019. Most cases were concentrated in Jiang’an (46), Xiling (37), Wuchang (11), Maojian (11), Qiaokou (10), and Gucheng (10), accounting for 45.45% (125/275) of cases during 2005–2019 (Fig. 4c, d e). Thirty-three counties reported imported *P. falciparum* in 2005–2009; however, this expanded to 66 counties during 2010–2014 and 71 counties during 2015–2019. Most cases were reported in Dongxihu (93), Xiling (66), Wuchang (63), Qiaokou (35), and Xialu (32), accounting for 33.37% of cases (289/866) (Fig. 4f, g, h).

### Region of infection acquisition

During 2005–2019, 1,141 imported cases, including 275 *P. vivax* cases and 866 *P. falciparum* cases, were reported in Hubei province. Two *P. vivax* cases and six *P. falciparum* cases were acquired overseas, which no country of origin information. Eight imported *P. vivax* cases were originated from other provinces in China. A total of 1125 imported *P. vivax* and *P. falciparum* cases from 47 other nations were detected, including 860 *P. falciparum* cases and 265 *P. vivax* cases. Of the 265 *P. vivax* cases, 145 (54.72%) were from nine Asian countries, such as Myanmar (77) and Laos (3), which are neighboring countries of China in Southeast Asia in which malaria remains endemic [1]. The majority of these cases were reported in May to September. A total of 115 *P. vivax* cases (43.40%) were imported from 20 countries in Africa, of which Ethiopia was the major source. The number of *P. vivax* cases that originated from Ethiopia rose from zero during 2005–2009, to 25 during 2010–2014, to 43 during 2015–2019.

**Fig. 5:**
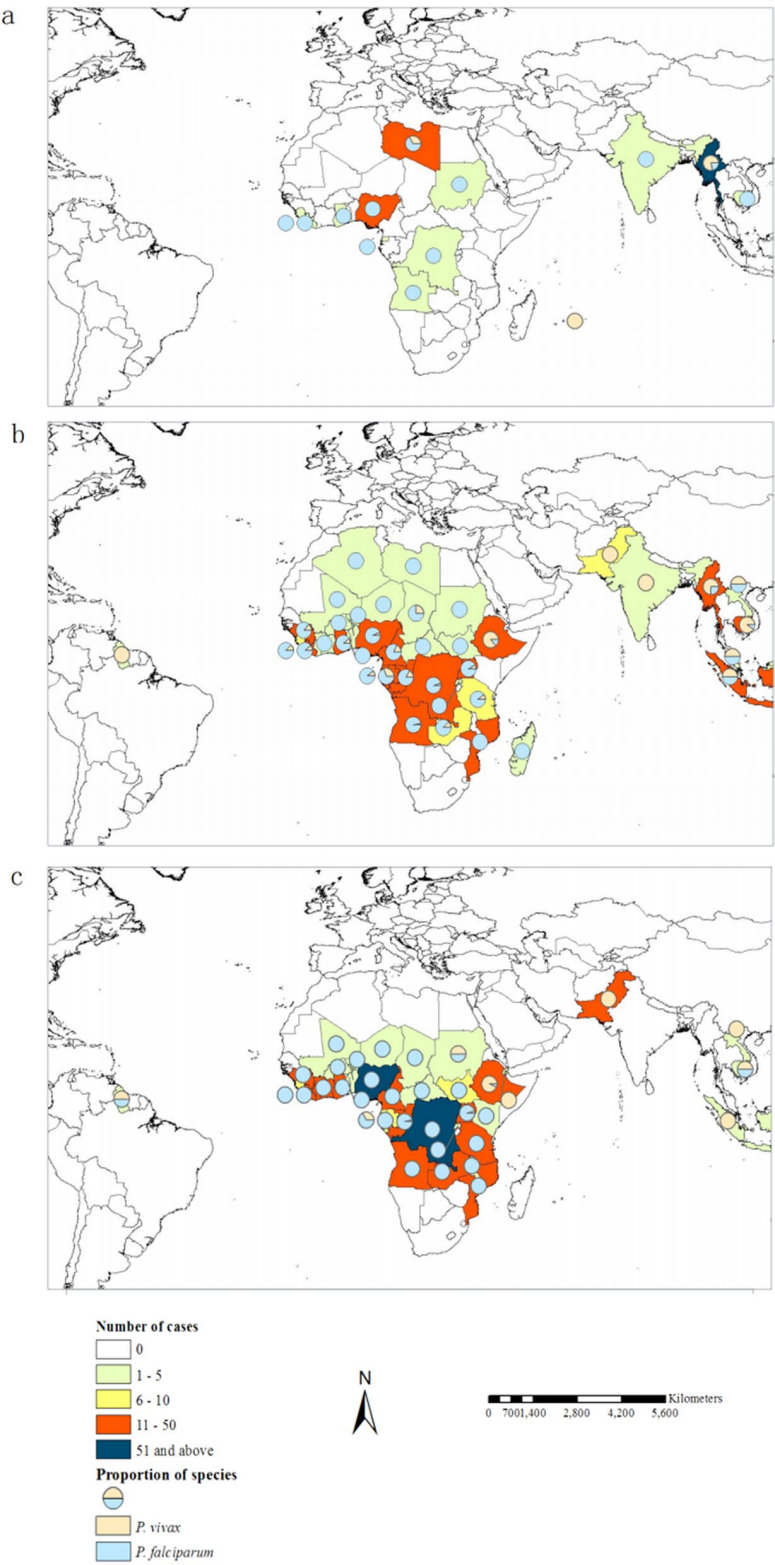
Potential sources of *Plasmodium vivax* and *P. falciparum* malaria imported into Hubei province, 2005–2019. **a** Intensified control stage (2005–2009). **b** Elimination stage I (2010–2014).** c** Elimination stage II (2015–2019)

*Plasmodium falciparum* cases originated from 32 countries in Africa (820/860, 95.35%). Congo-Kinshasa, Nigeria, Angola, Equatorial Guinea, and Liberia were the major countries of origin. Both *P. vivax* and *P. falciparum* cases from Africa displayed an increasing trend during the three consecutive stages. The number of African countries where *P. falciparum* originated rose from 9 during 2005–2009, to 30 during 2010–2014, to 29 during 2015–2019. In contrast, *P. vivax* and *P. falciparum* cases imported from Asia showed a decreasing trend (Fig. 5).

## Discussion

This study analysed 15 years of longitudinal surveillance data to investigate the proportion of imported vs. indigenous *P. vivax* and *P. falciparum* malaria cases in Hubei province, China, from the control stage to the elimination stages. Indigenous *P. vivax* malaria decreased markedly and has been eliminated in Hubei since 2013. However, imported *P. vivax* and *P. falciparum* malaria cases constituted 1.70% (116/6841) of all cases in the control stage, and had increased to 82.79% (534/645) in the elimination stage II.

Indigenous *Plasmodium vivax* malaria cases presented obvious seasonal fluctuations, and relatively more cases of indigenous malaria were reported from May to September. Imported *P. vivax* and *P. falciparum* malaria cases displayed seasonality, but the occurrence of peaks was not similar in the three stages: imported cases were observed in overseas workers who usually returned to Hubei for the Chinese Spring Festival (in January or February) and the busy farming season (from May to September). During the three stages, 80 *P. vivax* cases, imported from neighbouring countries in Southeast Asia (Myanmar, Laos), showed a seasonal pattern (May to September) that was similar to the historical indigenous *P. vivax* malaria cases in Hubei Province [8]. Since *An. sinensis* still existed in Hubei province, which can transmit *P. vivax* malaria [21]. All imported malaria cases were appropriately treated with antimalarial drugs. *P. vivax* cases received radical therapy, which included an anti-blood stage drug and primaquine as an anti-hypnozoite according to the national treatment guidelines in Hubei [22]. Patient’s compliance to complete the treatment course is key to the success of anti-relapse therapy. It is, therefore, necessary to strengthen compliance to standard malaria treatment so as to prevent the reestablishment of malaria transmission given the existing Anopheles vectors in Hubei.

Demographic characteristics differed between indigenous *P. vivax* malaria cases and imported *P. vivax* and *P. falciparum* malaria cases. The majority of imported *P. vivax* and *P. falciparum* malaria cases in 2005–2019 were observed among males aged 21–60 years. These results were consistent with observations made by several other research studies [23–25]. Along with the increased overseas business investment in recent decades [23], an increasing number of young male adults are affected because of work-related travel to highly endemic areas of Africa or Southeast Asia [26]. Most overseas work among this cohort is in infrastructural development and mineral mining, which are settings at high risk of malaria. Furthermore, information on the effects of malaria infection or protection against mosquitoes is usually lacking in these work environments [9]. Therefore, providing further information on the risks and effects of malaria as well as the measures to protect oneself is important in reducing the rate of malaria infection among migrant workers.

In the control stage (2005–2009), 77.76% of all indigenous *P. vivax* cases were concentrated in five counties (Xiangzhou, Zaoyang, Guangshui, Laohekou, and Zengdu). These were areas historically endemic for *P. vivax* malaria and have been receptive to transmission over the last several decades [27]. The sharp reduction in the number of indigenous *P. vivax* cases could be attributed to the implementation of malaria control measures for early detection of malaria, treatment of malaria cases, vector control in high-risk areas, and health education in high-risk populations [5, 28]. Different from indigenous *P. vivax* malaria, most imported *P. vivax* and *P. falciparum* cases were reported in counties belonging to several cities, such as Wuhan City (included Jiang’an, Wuchang, and Dongxihu counties), Yichang City (included Xilin county), and Huangshi City (included Xialu county), which have infectious disease hospitals with better medical treatment or large enterprises with more overseas job opportunities [29].

The number of regions affected by imported *P. falciparum* malaria increased markedly in Hubei in 2005–2019. *Plasmodium falciparum* infection can develop into severe malaria, which is related to a high case-fatality rate [11, 15, 30]. Cases of *P. falciparum* associated with death were reported in Hubei during this 15-year period [31, 32]. Therefore, the capabilities of early detection and appropriate treatment of *P. falciparum* malaria should be promoted at all levels of medical institutions in Hubei province. Cooperation should be encouraged among the health sector, the tourism sector, the commercial sector, the education sector and the Customs Department to facilitate early detection of imported malaria [33]. Furthermore, the 1-3-7 malaria case management model needs to be followed.

Of the total number of malaria cases, 54.72% of *P. vivax* cases were imported from Southeast Asia, whereas 95.35% of *P. falciparum* cases originated from Africa. From the control stage (2005–2009) to elimination stages I (2010–2014) and II (2015–2019), the reported malaria cases imported from Southeast Asia had a declining trend, which is contrary to the upward trend from Africa to Hubei. The number of reported malaria cases originating from Southeast Asia may have decreased in China due to the improvement of the malaria situation in the region in the last decade [7]. The “1-3-7” strategy has been adapted in some countries in Southeast Asia and Africa, which effectively reduce the risk of transmission [34–36]. Since 2010, Africa has become the main origin of imported malaria cases in Hubei, which was identical to the trend in the whole of China [7, 37], which might be due to the increasing investments and workers from China [11]. This suggests that medical staff should pay more attention to travel history, particularly to individuals coming from Africa and Southeast Asia [10].

This study had some limitations. First, cases with unclassified *Plasmodium* species existed during 2005–2010, which might have had an impact on the quantity and geographical distribution of indigenous *P. vivax* malaria. Second, the origins of the imported case may not be accurately determined because more travel destinations may exist. Third, the major contributors of malaria importation were not analysed in this study. Future studies should investigate the contribution of these factors to malaria importation in Hubei province, China.

## Conclusions

The current results suggested that some key comprehensive measures are required to maintain a sensitive surveillance system for imported malaria cases. These include a focus on pre-travel health education on malaria for migrant workers, promoting of the capabilities of medical institutions at all levels in Hubei province for early detection, rapid diagnosis, and appropriate treatment of malaria, and a more efficient multi-sector co-operation mechanism for imported malaria control, to reduce the potential risk of re-introduction.

## Data Availability

The datasets used and/or analysed during the current study are available from the corresponding authors on reasonable request.
